# Current opinions on large cellular models

**DOI:** 10.1002/qub2.65

**Published:** 2024-07-01

**Authors:** Minsheng Hao, Lei Wei, Fan Yang, Jianhua Yao, Christina V. Theodoris, Bo Wang, Xin Li, Ge Yang, Xuegong Zhang

**Affiliations:** ^1^ MOE Key Laboratory of Bioinformatics and Bioinformatics Division BNRIST Department of Automation Tsinghua University Beijing China; ^2^ AI Lab Tencent Shenzhen China; ^3^ Gladstone Institutes and University of California San Francisco California USA; ^4^ Department of Computer Science University of Toronto Toronto Ontario Canada; ^5^ State Key Laboratory of Stem Cell and Reproductive Biology Institute of Zoology Chinese Academy of Sciences Beijing China; ^6^ State Key Laboratory of Multimodal Artificial Intelligence Systems Institute of Automation Chinese Academy of Sciences Beijing China; ^7^ School of Life Sciences and School of Medicine Center for Synthetic and Systems Biology Tsinghua University Beijing China

**Keywords:** large cellular models, large language models, scBERT, Geneformer, scGPT, scFoundation, GeneCompass, single‐cell transcriptomics

## 1 INTRODUCTION

Large language models (LLMs) have made breakthroughs in natural language processing (NLP) and understanding, and have brought revolutions in many other fields [1, 2, 3, 4]. Inspired by those successes, several large cellular models (LCMs) adopting similar structures of LLMs have been developed for single‐cell transcriptomics, including (but not limited to) scBERT [5], Geneformer [6], scGPT [7], scFoundation [8], and GeneCompass [9]. The practices of these models have shown LCMs’ power and potential in various biological tasks and illustrated the possibilities of revolutionizing future biological studies by LCMs.

**FIGURE 1:**
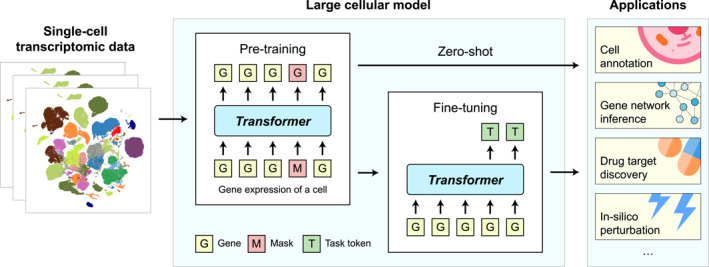
Schematic diagram of large cellular models.

Figure 1 shows a general framework of LCMs. The models are **pretrained** on massive single‐cell transcriptomic datasets of multiple human tissues and organs or even including data of other species. Each cell in the dataset is characterized by gene expression values and serves as a training sample. Similar to LLMs, LCMs commonly use the **transformer** [10] architecture and are generally pretrained using a self‐supervised strategy of either **masked modeling** [11] or **autoregressive generative modeling** [12] techniques. In masked modeling, we first randomly mask some genes’ expression in a cell, and then let the model convert the genes and their expression values into **embeddings**, and predict the masked gene expressions based on observed genes as the contexts. The learnable parameters in LCMs are optimized to minimize the **loss function** between predicted and ground truth gene expression values. Usually, the sizes of the models and the numbers of parameters are very large, currently at levels from tens of millions to billions. That is why these models are called large models. It has been shown that given sufficient data, the models can benefit from larger sizes in their pretraining performances on both the natural language data [1] and single cell data [8]. The pretraining procedure enables LCMs to learn about gene relationships as well as cell characteristics. After pretraining, LCMs can be applied in both **zero‐shot** and **fine‐tuning** paradigms, solving an array of downstream biological tasks including cell annotation, gene network inference, drug target discovery, in‐silico perturbation, etc.

**BOX 1 Glossary of some machine learning concepts**

**Pretraining**: Pretraining is a process where a model learns from large amounts of data before being used on specific tasks. This initial phase allows the model to capture general patterns and semantics, enhancing its performance and adaptability across various specific applications.

**Self‐supervised learning**: Self‐supervised learning is a strategy of machine learning that does not use extra labels to supervise the learning procedure, but instead uses the task of predicting certain parts of the data from other parts as the objective of learning. This approach eliminates the need for labeled data, as the model generates its own supervision signal during training.

**Embedding**: Embedding in the general sense is to represent objects such as text, images, genes, or amino acids using mathematical vectors in certain continuous space where the locations of the vectors represent meanings or semantics of the objects with regard to their relations. In LCMs, embeddings are compact representations of the transcriptomic data in a mathematical space, encoding gene symbols and expression value information. LCMs can also obtain embeddings for cells and cell states.

**Loss function**: Loss function is one of the key elements in the design of a machine learning model and algorithm. It quantifies the difference between predicted and true values, guiding the model to adjust its parameters to minimize this difference.

**Transformer**: Transformer is a neural network architecture that uses self‐attention mechanisms to capture relationships between embeddings of the input objects (tokens). It is widely used in large artificial intelligence (AI) models nowadays due to its scalability and effectiveness.

**Masked modeling**: In masked modeling, parts of the input data are replaced by a specific mask embedding, and the model learns to predict these parts based on the remaining information. It is like a “cloze test”: When the model learns to predict the masked data, it “understands” the data.

**Autoregressive generative modeling**: In autoregressive generative modeling, the model generates new data points given the preceding ones in a sequence. In LCMs, gene expression rank can be regarded as sequential information.

**Zero‐shot learning**: Zero‐shot learning is a machine‐learning paradigm where a model is trained to perform tasks it has not been explicitly trained on. In LCMs, it usually refers to extracting cell or gene embeddings for downstream tasks from the pretrained model without the need for extra training for specific tasks.

**Fine‐tuning**: Fine‐tuning is a process where a pretrained model is further trained on a specific task or dataset to improve its performance. This is particularly useful when the pretrained model has learned useful representations that can be adapted to the target task, saving time and computational resources.

Large AI models in biological fields such as LCMs on single‐cell transcriptomics data have begun to flourish in the past 2 years. At this critical time for great advancement and rapid development, *Quantitative Biology* took the honor to interview some of the key authors of the most recent representative works of LCM. The authors we interviewed and the models in the order of their publication or preprint release are: Fan Yang and Jianhua Yao (FY&JY) of scBERT [5], Christina V. Theodoris (CT) of Geneformer [6], Bo Wang (BW) of scGPT [7], Xuegong Zhang (XZ) of scFoundation [8], and Xin Li and Ge Yang (XL&GY) of GeneCompass [9]. We asked their opinions on a few common questions and questions specific to their models. The questions range from brief introductions to their models, the advantages and challenges, characteristic biological applications, and their perspectives for the future of the field. We collect these interviews upon all authors’ consent to form this special article to reflect the current opinions on this exciting topic. These opinions shed light on the transformative impact of LCMs on biological research and provide a glimpse into a future where AI and life sciences converge to answer the key questions about life.

## 2 INTERVIEWS AND OPINIONS

### 2.1 Questions for all authors

QB: *Can you briefly describe to our readers what your model is and what it can be used for?*

- FY&JY: scBERT [5] is a pretrained language model designed to transform single‐cell transcriptomic data into a universal embedding. This transformer‐based model is trained using the BERT [11] paradigm and could be utilized for various applications, including cell type annotation, discovery of novel cell types, and identification of novel marker genes.
- CT: Geneformer [6] is a foundational deep learning model pretrained on a large‐scale corpus of ∼30 million single‐cell transcriptomes to enable context‐specific predictions in settings with limited data in network biology through transfer learning. With both zero‐shot learning and fine‐tuning with limited data, Geneformer consistently boosted predictive accuracy in a diverse panel of biologically meaningful downstream tasks. We demonstrated Geneformer’s ability to make new biological insights including discovering a novel transcription factor in cardiomyocytes through zero‐shot learning and revealing candidate therapeutic targets for cardiomyopathy using limited patient data, both of which we experimentally validated with functional assays in cells. Geneformer’s fundamental understanding of gene network dynamics can now be democratized to a vast array of downstream tasks to accelerate discovery of key network regulators and candidate therapeutic targets.
- BW: scGPT [7] is a single‐cell foundation model pretrained on 33 million human cells. Inspired by LLMs, scGPT, as a foundation model, aims to first learn single‐cell biology from the large‐scale diverse pretraining dataset of human cells, and then effectively transfer the knowledge to a variety of downstream tasks. scGPT leverages a transformer backbone with attention to capture the intricate interconnectivity between genes. More importantly, scGPT designs a special attention mechanism with cell prompts and gene prompts that enable generative training with the nonsequential scRNA‐seq data in an autoregressive manner. The pretrained scGPT model demonstrates robust extrapolation to new datasets, accurately clustering cell types in zero‐shot experiments and aligning with known gene networks. Through fine‐tuning, its knowledge is transferable to various tasks, consistently outperforming specialized models in cell type annotation and perturbation prediction tasks.
- XZ: scFoundation [8] is a pretrained model on single‐cell transcriptomics data and is on a large scale in terms of parameter size, gene dimensionality, and size of training data. Benefiting from the model architecture design and training strategy, it can provide valuable embeddings for both single‐cell and bulk analyses. These embeddings can be applied to a variety of cell‐level tasks such as enhancing gene expression, annotating cell types, and predicting drug responses in tissues and individual cells. Additionally, scFoundation can generate gene‐level embeddings useful for inferring gene networks and predicting the effects of perturbations on single cells.
- XL&GY: GeneCompass [9] is a knowledge‐informed cross‐species foundation model, pretrained on over 120 million human and mouse single‐cell transcriptomes. Four kinds of prior knowledge, that is, promoter sequence, gene family, gene regulation networks (GRN), and co‐expression relationship are integrated into GeneCompass by encoding them into the input. GeneCompass can promote a wide range of applications across the biological field, including cross‐species cell type annotation, GRN prediction, drug dose‐response prediction, and perturbation prediction. Moreover, GeneCompass can accelerate the discovery of critical cell fate regulators by in silico gene perturbation in the high‐dimensional embedding space.

### 2.2 Interview on scBERT

1. QB: *Is scBERT the first transformer‐like model developed for single‐cell RNA‐seq data? Can you tell our readers about how you started the project and what major challenges you had met in the work?*
  FY&JY: We started scBERT project in early 2021, and released the code and preprint paper in December 2021. To our knowledge, scBERT is the first transformer‐like model for single‐cell RNA‐seq data. Inspired by the success of BERT [11] paradigm in NLP, we ventured to apply BERT on single‐cell RNA‐seq data. This pioneering work presented several challenges, with the primary one being the transformation of single‐cell RNA‐seq data (typically in count‐matrix format) into input embedding for the transformer. After a thorough examination of the nature of scRNA‐data and discussions with NLP experts, we designed gene embedding and expression embedding, analogous to position embedding and word embedding in NLP. The successful implementation of these embeddings has since inspired the design of several subsequent transformer‐based models. Other challenges included collecting a large amount of suitable training data and how to effectively conduct self‐supervised pretraining.
2. QB: *What do you think are the key technical challenges for AI models to be expanded or adopted for life science tasks?*
  FY&JY: I think the key technical challenges for AI to be expanded or adopted for life science tasks lie in defining the problem and formulating a biologically valuable problem into an object optimizable by AI algorithms. Another important technical challenge is how to build a high‐performance model based on extremely scarce experimental data. I think it is a common scenario in life science.
3. QB: *scBERT was built 3 years ago. Can you update on the research in your labs since then?*
  FY&JY: We have since explored AI applications for spatial omics and single‐cell proteomics. In the aspect of spatial omics, we developed a cell type annotation algorithm [13], and a microenvironment analysis tool [14]. As for single‐cell proteomics, we developed a general embedding framework [15] and a deconvolution algorithm [16]. All of our works focus on the central dogma and its potential biological applications.
4. QB: *What is your vision on the potential application of AI models in biological studies?*
  FY&JY: AI could facilitate various aspects of biological studies, from understanding and discovery to creation. Analyzing single‐cell multi‐omics data allows us to understand the interplay between DNA, RNA, and protein in individual cells from a systematic perspective. Linking this data (optionally other modality data) to the phenotype such as patient‐level label can help us understand disease process and discover new targets for novel therapies. By modeling atom‐level interactions between proteins and other molecules, we can de novo design (creating) novel protein binding drugs. In essence, we can use AI to help us understand the nature of our lives and improve the quality of our life.
5. QB: *The fields of both AI and biology are developing very quickly, with technologies updated or even revolutionized in an unprecedented speed. What is your perspective on the recent future of AI applications in biological research?*
  FY&JY: As we all know that AlphaFold2 has revolutionized the structure biology by relatively accurately predicting protein structure from sequence. More models have been emerged to predict the structure of complex where proteins interact with other molecules, such as nuclei acids, chemical drugs, and covalent modifications. In the recent future, I think AI for protein complex analysis, combined with cellular context provided by single‐cell multi‐omics is expected to bring valuable applications for the biological research.

### 2.3 Interview on Geneformer

1 QB: *What was the major biological discoveries from your application of Geneformer on real biological questions? Can those discoveries also be found with more traditional statistical or machine‐learning methods?*
  CT: We demonstrated Geneformer’s ability to enable predictions in a diverse range of biological settings with both zero‐shot learning and fine‐tuning, including in gene network dynamics (e.g., predicting gene network centrality, transcription factor targets and transcription factor cooperativity), chromatin dynamics (e.g., predicting bivalent promoters and transcription factor regulatory range), dynamic cell trajectories (e.g., in silico reprogramming and differentiation), and disease‐dependent dysregulation (e.g., determining disease‐driving genes and candidate therapeutic targets). We applied Geneformer to a broad range of tissues, diseases, and developmental stages to confirm the generalizability of its fundamental knowledge. We also compared Geneformer to alternative machine learning approaches such as random forest, support vector machines, and logistic regression and found that Geneformer consistently boosted predictive accuracy.
    In terms of novel discoveries, we designed an in silico perturbation approach that discovered a novel transcription factor in cardiomyocytes through zero‐shot learning that we confirmed experimentally was critical to the ability of the cells to generate contractile force. It was exciting to us that Geneformer was able to discover this novel regulator despite decades of prior research on cardiomyocytes and that the predictions were validated to have a true biological effect in cells.
  We then extended our approach to an in silico treatment strategy, which discovered novel therapeutic targets in cardiomyocytes that significantly improved the ability of the cells to generate contractile force in an induced pluripotent stem cell disease model of cardiomyopathy. We were excited that the model was able to predict novel therapeutic targets that had a true biological impact on the phenotype in cells and are looking forward to seeing how others use Geneformer to drive discoveries in other diseases and biological settings in the future.
2 QB: *You are a scientist working on cardiovascular diseases, can you give some scenarios in cardiovascular research that you expect AI especially LCMs will make major roles?*
  CT: More broadly than cardiovascular disease, one of the major obstacles in biology is that it is infeasible and cost‐prohibitive to test all of the astronomical number of perturbations one could test in wet lab experiments to discover network regulators and therapeutic targets. One of the major promises of AI is the ability to efficiently computationally prioritize downstream experiments in an unbiased, data‐driven manner. Furthermore, by employing a closed‐loop approach, data from the prioritized downstream experiments in the wet lab can provide feedback to the model about where its predictions were correct and where they failed, thereby continuously improving the models’ predictions with real‐world data.
3. QB: *In the Discussion of your article, you predicted that “as the amount of publicly available transcriptomic data continues to expand, future models pretrained on even larger‐scale corpuses may open opportunities to achieve meaningful predictions in even more elusive tasks with increasingly limited task‐specific data,” can you give some examples on possible task that may fall into this category?*
  CT: We pretrained Geneformer in June 2021 and since then there has been a rapid expansion in the amount and diversity of single‐cell transcriptomic data available in the public domain. One of the most difficult tasks we tested in the Geneformer manuscript was predicting whether transcription factors act in short or long range with their targets. This is an especially difficult task for the model to predict using only transcriptomic data without information about genomic distance. However, the model was able to predict to a degree this higher‐order attribute of transcription factors while more traditional machine learning methods had random predictions. As models are pretrained on even greater amounts of data, they may gain further fundamental knowledge that allows them to better predict these higher‐order characteristics of genes. Additionally, Geneformer was able to predict network centrality of genes with as few as ∼800 task‐specific cells. As the model gains further fundamental knowledge during larger‐scale pretraining, these tasks could be accomplished with even fewer task‐specific cells or even by zero‐shot learning with no fine‐tuning data.
4. QB: *What barriers does your lab face in developing AI models for biology?*
  CT: The largest barrier continues to be the access to sufficient GPU compute resources to train the types of models we are interested in, which is something most academic settings are facing compared to resources available to companies in industry. Another major barrier is unifying data that is deposited in the public domain with extremely variable formats and many times little to no information about how the data was previously processed or relevant metadata such as whether the samples were derived from healthy or disease patients, etc. As we recognize the promise of AI in biomedical research, it will be important to develop systems to structure data as AI‐ready to gain the most out of the enormous worldwide funds invested in biological research. The CELLxGENE [17] is a database of this kind that allows efficient Application Programming Interface (API)‐based access to large amounts of single‐cell data, providing an example for others to follow for other types of biological data.
5. QB: *It has been more than 1 year since your work was published, can you brief to our readers the on‐going research in your lab that is built on or related with Geneformer or other large cell models? Nowadays many biologists are interested to bringing AI especially large models into their research, do you have any suggestions to them on the possible routes to make their efforts smoother and/or possible pitfalls they may need to be careful to?*
  CT: Our lab leverages AI and experimental genomics to address major challenges in gene network biology. Part of our lab is focused on developing new AI models expanding our transfer learning approach to tackle new directions such as how gene networks affect cell interactions through space and time. The other part of our lab is applying these models to investigate basic unanswered questions in gene regulation and to identify network‐correcting therapeutics for human disease. We emphasize strong collaboration between our computational and experimental members to promote a closed‐loop AI and experimental genomics strategy to accelerate our discoveries.
    In terms of integrating AI into biological research, one common pitfall is that users apply default hyperparameters to all of their tasks of interest, whereas hyperparameter tuning is very important and can be the difference between a model learning nothing at all or having a near perfect predictive accuracy. Other suggestions include ensuring that data are balanced between other potential confounding attributes and collecting enough data to split into separate training, validation, and test sets by sample/individual as opposed to subsampling cells from all conditions for all splits. Of note, if a validation set is used to optimize hyperparameters, a separate held‐out test set is used to confirm the generalizability of the best model to unseen data.

6. QB: *Both the fields of AI and of biology are developing very quickly, with technologies updated or even revolutionized in an unprecedented speed. What is your perspective on the recent future of AI applications in biological and medical research?*
  CT: This is an extremely exciting time in the fields of both AI and biology. With the current boom of large‐scale biological data, we are now entering the realm where large‐scale AI models can potentially be trained to have a fundamental understanding of biology. As foundation model approaches are adopted by the biological research community, there is also an opportunity to drive innovation in AI based on the unique features of biological systems, such as the need to abide by the laws of physics that constrain the protein structures that are physically possible, etc.

### 2.4 Interview on scGPT

1. QB: *There have been high expectations on the application of large cell models in biological studies, but there are also suspicions on the necessity of large models. There are arguments that the superior performance of LCMs on many single‐cell analysis tasks can also be reached by carefully designed methods for those specific tasks, but those methods can be lighter in terms of the data and computation cost. What is your opinion on such suspicions?*
  BW: It is without a doubt that simpler models can be optimized to perform well on specific datasets for specific tasks. There are two limitations with the “small model” approach that LCMs can complement.
    Firstly, the modeling capability of small models is limited by parameter size. Because of this limitation, most of the current analysis approaches rely heavily on variable gene selection or other pre‐processing steps to reduce the input size and heterogeneity. On the other hand, LCMs use attention to capture gene‐level interactions from the entire genome, presenting a more complete picture of gene interactions that could potentially aid broader hypothesis generation.
    Secondly, observations from biological experiments are noisy. Small models often face difficulty generalizing to unseen datasets or experiment conditions, and are prone to overfitting to noise from the experiment at hand. Having the model learn cell representations from large‐scale heterogeneous data helps discern biological signals from noise, presenting a more unbiased view of underlying biology.

2. QB: *Current LCMs are all based on the basic transformer structure originally developed for tasks in NLP. Different LCMs used different ways to adopt the basic structure to be applicable on biological data. Based on your experience in developing scGPT, what are the key challenges in adopting LLMs for LCMs? Do you perceive that there is a need or possibility to design fundamentally different structures specifically for biological data and tasks?*
  BW: The key challenge that we faced when developing scGPT was how to best adapt generative pretraining used in LLMs to LCMs, given the nonsequential nature of single‐cell data. On a high‐level, autoregressive training and generation align with the idea of capturing a cascade of gene interactions and predicting cellular responses to perturbations. Alternative architectures include BERT and diffusion models, which are also worth exploring without assuming sequences.
3. QB: *Some bioinformaticians are saying that with the involvement of large models, the cost for doing bioinformatics research has boomed comparing with the “good old days” when most research can be done with relatively simpler mathematical models and simpler algorithms based on smaller data. Do you agree on this argument, or do you have any suggestions for bioinformatics labs with less computation resources on how they may benefit from the advancement of current AI?*
  BW: We aim to develop tools that benefit the community and assist biologists in their day‐to‐day work. For scGPT specifically, we hosted the model on scGPT Hub where biologists can finetune the model with a dataset upload. It is important to consider accessibility when designing new models, and we envision more cloud‐based platforms with reduced compute barriers readily available to biologists as we enter the LCM era.
4. QB: *Both the fields of AI and of biology are developing very quickly, with technologies updated or even revolutionized in an unprecedented speed. What is your perspective on the recent future of AI applications in biological and medical research?*
  BW: AI is a valuable tool that gives more modeling power to biologists to model the complex processes in biology. Recent predictive models in clinical research have benefited the day‐to‐day workflow in hospitals and clinics, raising the standards of patient care. We envision AI to be integrated in a similar manner for biological research, with biologists in the loop, to help them solve their problems more effectively and efficiently.

### 2.5 Interview on scFoundation

1. QB: *There are some misunderstandings in the community that LCMs are simply straightforward applications of transformers to single‐cell data. Based on your experience, what are the key challenges in designing a model that is suitable for cell data? What are the key solutions that distinguish scFoundation from other LCMs?*
  XZ: The key challenges of designing the model lie in handling the high dimensionality and high sparsity characteristic of scRNA‐seq data, and in eliminating technical noises from biological variations. Specifically, when modeling each cell as a sentence and each gene expression value as a word, the nearly 20,000 protein‐coding genes make the “sentence” exceptionally long, a scenario that traditional transformers struggle to handle. As for technical noise, scRNA‐seq data across different techniques and laboratories exhibit high variance in sequencing read depth.
    To address these challenges, scFoundation utilizes the scalable transformer‐based architecture xTrimoGene [18] and a novel read‐depth‐aware (RDA) pretraining task that builds on masked language modeling principles.
    The xTrimoGene architecture features an embedding module that converts continuous gene expression values into learnable high‐dimensional vectors without approximation and an asymmetric encoder–decoder structure tailored to efficiently learn the relationships between 20,000 genes while accommodating the high sparsity of single‐cell gene expression data.
    In RDA modeling, the task is to predict the masked gene expression in a cell using the context provided by other genes within the same cell, whether those gene expressions are with the original or lowered read‐depth. This approach not only captures gene–gene relationships but also harmonizes cells across different sequencing depths, featuring a unique design of scFoundation among other LCMs.

2. QB: *Many biologists are eager to introduce LCMs into their projects, what are the most typical ways for biologists or bioinformaticians to use scFoundation in their work? And what are the benefits they may expect from such applications?*
  XZ: To study the integration of LCMs such as scFoundation into biological research, it is helpful to categorize their applications into two main types: cell‐level tasks and gene‐level tasks. Cell‐level tasks typically focus on identifying characteristics of cells, such as cell type annotation or drug sensitivity prediction. Gene‐level tasks are more about understanding relationships between genes or predicting changes in gene expression, such as through gene network inference or gene perturbation prediction.
    For cell‐level tasks, a common approach with scFoundation is to utilize the model’s encoder to obtain read‐depth enhanced embeddings. These embeddings can be quickly generated and applied to various downstream tasks, providing rich latent representations of cells enhanced for read depth in just minutes, without the need for extensive computational resources or time‐consuming fine‐tuning. This process effectively decouples the generation of embeddings from downstream analysis, affording significant flexibility in the application of subsequent models.
    For gene‐level tasks, the typical usage involves extracting gene embeddings from the model’s decoder. Importantly, scFoundation provides context embeddings for all genes within each cell, enabling the construction of cell‐specific gene co‐expression networks. This capability is invaluable for users looking to develop more sophisticated and accurate algorithms. For instance, these context embeddings can serve as inputs to perturbation prediction models such as GEARS [19], enhancing the accuracy of their predictions. This dual‐application approach allows users to leverage scFoundation to advance their research significantly, both in terms of efficiency and scientific insight.

3. QB: *Some bioinformaticians are saying that with the involvement of large models, the cost for doing bioinformatics research has boomed comparing with the “good old days” when most research can be done with relatively simpler mathematical models and simpler algorithms based on smaller data. Do you agree on this argument, or do you have any suggestions for bioinformatics labs with less computation resources on how they may benefit from the advancement of current AI?*
  XZ: We fully understand the concerns regarding the cost comparisons between LCMs and classical bioinformatics methods. However, we believe that these two approaches are not competitors but rather complementary. For example, in our scFoundation work, we demonstrated that while the scFoundation model excels in performance when fine‐tuned for specific tasks, it can also be effectively combined with existing models to enhance overall performance. This integration highlights a future direction for incorporating large‐scale foundation models into users’ workflows, thus alleviating the heavy computational demands of training.
    To assist labs with limited computational resources, we advocate for open sourcing both the model code and weights, as exemplified by scFoundation and other LCMs available on GitHub and/or Hugging Face Model Hub. Additionally, we have developed an online web service and an API for scFoundation, which allows users to directly utilize pretrained embeddings for subsequent tasks. This API offers a more accessible and practical solution for individual labs, eliminating the need for dataset‐specific re‐training or fine‐tuning.

4. QB: *In the NLP field, people say that they observed a “scaling law” that the performance of the model always improves when more data and larger models are involved. Have you observed the same law on LCM? Is there a way to determine the “proper” model scale?*
  XZ: Indeed, we have observed a scaling law similar to that in NLP in our work. We pretrained scFoundation models with 3, 10, and 100 million parameters, and noted that the models’ capacity to accurately predict masked cell gene expression increases with size. This suggests that even larger scFoundation models could potentially offer even greater predictive performance, indicating that we have not yet reached the upper limits of the model scale.
    Determining the “proper” model scale involves several factors. Firstly, amassing as many single‐cell datasets as possible is crucial for scaling up models effectively. Our findings in the xTrimoGene paper [18] confirm that larger datasets significantly enhance performance. Secondly, the model architecture itself is pivotal; given the unique loss functions and data modalities in LCMs compared to NLP, it is vital to design pretraining models that maintain the scaling law.
    Once these factors are addressed, the appropriate size for LCMs can be gauged by referencing NLP models trained with a similar volume of data tokens. Finally, deployment costs must also be considered. As we are still at the starting point of LCM development, it is essential to balance the cost implications for users against their expectations. In the case of scFoundation, we strive to maximize model size while ensuring it remains manageable, such as being trainable on a single A100 GPU and deployable on more commonly available GPUs such as the RTX4090. This approach helps us to maintain a balance between computational power and accessibility.

5. QB: *Given that the scFoundation retains all genes for training, could the model be expanded to encompass multi‐omics data? This extension might offer a pathway to gain deeper insights into cellular biological processes*.
  XZ: Integrating multi‐omics data indeed can offer a holistic perspective on cellular states. To extend the capabilities of scFoundation to encompass multi‐omics data, such as ATAC/RNA integration, we could implement several possible strategies. One possible approach involves designing tasks that leverage both gene expression and ATAC‐seq data. For instance, we could develop models to predict gene expression values based on ATAC‐seq context and vice versa. Specifically, when predicting ATAC‐seq information, we could enhance the existing scFoundation architecture by incorporating additional transformer blocks tailored for each gene context embedding derived from the pretrained scFoundation. These embeddings would then be processed by the new transformers to predict the chromatin accessibility peaks associated with specific gene regions. Considering the potentially vast number of accessible peaks, it might be prudent to explore advanced transformer architectures to efficiently handle the data.
6. QB: *The world is witnessing the rapid advancement in the AI field is the high‐fidelity generation of multi‐modality information such as texts, images, voices, and videos. Do you think these technologies will soon also be adopted in biological studies?*
  XZ: Generative learning is one important paradigm in current large AI models and has shown great success in many tasks. Encouraged by the success of scFoundation, we have developed a new model scMulan [20] that uses the pure generative paradigm to learn the cell language that includes both gene expression data and various types of metadata [20]. Primary experiments have already shown its advantage in certain tasks and its power in conditional generation of synthetic single‐cell transcriptomics data that can carry real biological information. It has shown high potential for conducting virtual cellular experiments such as in silico perturbation and generation of cells along a trajectory of biological events. Learning and generation of data cross multiple biological modalities is a topic that many labs are working on. We believe that the technology advancement in multi‐media data understanding and generation will for sure provide solutions or hints for solutions. However, it is hard to predict how soon this will succeed. A fundamental difference between the biological scenario and the multimedia scenario is that people are experts in understanding multimedia data and their underlying meanings, but are still far from really understanding the biological data and their underlying meanings in many modalities. It is a promising direction to explore, but finding feasible angles that are compatible with current availability of technology, data and knowledge is vital.

### 2.6 Interview on GeneCompass

1. QB: *A unique feature of GeneCompass comparing to other published LCMs is that it was trained with data from both human and mouse. What do you think is the benefit of this setting? What downstream tasks are there that can only be fulfilled with GeneCompass but not LCMs pretrained only on human data?*
  XL&GY: As mentioned in the background, massive single‐cell data is essential to pretrain a LCM. Data with more diversity and larger volume is believed to build a better performance. Compared with LCMs pretrained only on human data, GeneCompass is the one that pretrained on the most volume of data, over 120 million cells, which made it outperform some earlier LCMs on several downstream tasks. The intrinsic biological principle is the GRN conservation between human and mouse. The learned latent gene relationship of a species would be transferred to the other one through homological gene mapping, which would be of vital importance for those downstream tasks that require ChIP‐seq data. If only on human data, cross‐species cell type annotation is one of the downstream tasks that cannot be fulfilled. Depending on pretrained GeneCompass, we utilized mouse cell type as a reference to annotate human cells on seven paired datasets from four distinct organs (retina, brain, pancreas, and testis). A 7.5% improvement compared to CAME is observed in the Retina, demonstrating GeneCompass can compete with and even overperform the leading specialized cross‐species cell annotation tool.
2. QB: *A new feature of GeneCompass is the integration of knowledge in modeling the genes. Many people believe that integrating data and knowledge in designing AI systems is the future solution for complicated biological tasks. Can you give your insight on the possible strategies or approaches on this topic based on your practices?*
  XL&GY: Most of the existing LCMs are only driven by data through a self‐supervised learning paradigm. Introducing the accumulative prior knowledge can complement the life information that training data may not contain. The strategy that integrating knowledge should vary with its type. For GeneCompass, we integrated four types of knowledge including promoter sequence, gene family, GRN, and co‐expression relationship by encoding each into an embedding vector and concatenating them with the embedding of the single‐cell transcriptome. This is a strategy of integration at the input level. Moreover, there are also some other strategies such as knowledge‐guided pretraining task and knowledge‐supervised loss. The optimal strategy remains an area of ongoing research and will be the focus of our future work.
3. QB: *What are the key challenges in the development of the GeneCompass model and its pretraining method when you made the model for cross‐species data?*
  XL&GY: The key challenge for developing a cross‐species LCM is how to integrate single cell data from human and mouse whose genes are distinct from each other. Concatenating their gene list may be a naive and easily realized solution but cannot take advantage of the gene conservation. The super long gene list requires more computational power and memory space. To solve this problem, we designed a homologous alignment strategy that maps genes between human and mouse based on the homologous relationship. The homologous genes will share the same gene ID in our gene list. Furthermore, we encoded the human and mouse prior knowledge into a unified representation space to ensure semantic consistency across species. Our experimental results of comparing the homologous gene embedding similarities with nonhomologous ones verified the effectiveness of this strategy.
4. QB: *Your work has included examples of identifying key transcription factors. Since most available single‐cell data are static snapshots of the gene expression at the time when the sample is obtained, how do you think LCMs may help in understanding dynamic cellular processes such as cell‐state transition and identifying key factors that may drive such processes?*
  XL&GY: Like the LLMs in the general NLP domain, the basic philosophy of pretrained LCMs is that extensive training on diverse datasets collected from a wide range of sources helps the model learn a broad understanding of cell, context and life. Although most available single‐cell data are static snapshots, the wide range of pretrained data covers the different stages of cell transition. Depending on the pretrained LCMs, similar cell status data would have strong similarities in the encoded embedding space, which would help in understanding dynamic cellular processes. To identify the key factor, we conducted in silico gene perturbation to overexpress or knockout a gene to a certain expression level. By comparing the similarity of the perturbed cells with the original cells and the target cells, the potential key factors can be identified. The results from our experiments and Geneformer both verified the effectiveness of this method. Our wet experimental results also proved the partial function of the identified key factors. This is of great significance for the discovery of critical cell fate regulators and candidate drug targets.
5. QB: *Both the fields of AI and of biology are developing very quickly, with technologies updated or even revolutionized in an unprecedented speed. What is your perspective on the recent future of AI applications in biological and medical research?*
  XL&GY: I believe the cross‐discipline between AI and life science will drive remarkable breakthroughs in biological and medical research. Especially with the emergence of foundation models, LCMs can generalize well to new, unseen tasks without the need for specific task‐oriented training or fine‐tuned with relatively small datasets, making them versatile tools for a range of downstream applications. On one hand, the use of LCMs will significantly reduce the time and economic costs associated with biological and medical research. More and more in vivo and in vitro experiments will be simulated through LCMs, which would help improve the success of traditional wet experiment‐based tasks such as drug discovery, protein structure design, etc. On the other hand, there have emerged AI models at different levels of life process, such as the LCMs at the transcriptome level, AlphaFold and ESMFold at the protein level, and EVO at the DNA level. There also have been multimodal LLMs that can be used to understand phenotype data such as medical images. I believe that there will be a unified model to integrate biology and model the central dogma to build the complex relationships between genotype and phenotype of all lives.

## 3 CONCLUSION

This interview collected the current opinions on the development of LCMs and their applications from some of the leading teams in this field. The models we interviewed on have shown great success in many downstream biological tasks, but the whole field is still in its early development phase. It is too early to ask the question about what would be the best or converged model structure for single‐cell transcriptomics data. The authors’ descriptions provided insights on the distinctive features of different models, which can help readers to obtain an overall understanding of the field rather than random samples of individual models.

It is valuable that the authors shared their strategies and considerations for the key technical challenges in developing and adopting transformer‐based AI models for biological objects, such as transforming complex single‐cell data into AI‐compatible formats, handling high dimensionality and sparsity, and optimizing model performance with limited data and resources. They also shared strategies in model development and optimization, including the adaptation of general pretraining techniques to nonsequential single‐cell data and the design of models that can discern biological signals from noise. The opinions also include thinking and practices on the challenges in computational resources for downstream users and for the development of cloud‐based platforms to make large AI models more accessible to biologists. The authors also provided their perspectives and suggestions for better using LCMs in future biological investigations.

Compared to the field of AI in NLP and computer vision, the field of biology lacks systematic benchmarking datasets, tasks, and measurements for the development of machine learning technology. Each study finds its own data and design their own experiments to test their models and algorithms and to show usefulness of their methods. This situation is not ideal for the healthy development of the field. Christina V. Theodoris highlights this question and her opinion on it in a separate perspective article in this issue [21]. This is an important topic that can have significant influences in the evolution of AI methods for biological tasks. Biology is complicated and we cannot dream for any technology that can suddenly answer all major biological questions even if the technology has been proven successful in many other fields. Building a systematic test field at multiple levels for developing, evaluating, and selecting technologies that fit the nature of biological questions is a crucial yet under‐investigated topic in people’s efforts on AI for life sciences.

## AUTHOR CONTRIBUTIONS

**Xuegong Zhang**: Conceptualization; funding acquisition; project administration; writing – original draft; writing – review & editing. **Minsheng Hao**: Writing – original draft; writing – review & editing. **Lei Wei**: Writing – original draft; writing – review & editing. **Fan Yang**: Writing – original draft; writing – review & editing. **Jianhua Yao**: Writing – original draft; writing – review & editing. **Christina V. Theodoris**: Writing – original draft; writing – review & editing. **Bo Wang**: Writing – original draft; writing – review & editing. **Xin Li**: Writing – original draft; writing – review & editing. **Ge Yang**: Writing – original draft; writing – review & editing.

## CONFLICT OF INTEREST STATEMENT

The authors declare no conflicts of interest.

## Data Availability

Data sharing is not applicable to this article as no new data were created or analyzed in this study.
